# Trichoderma combined with palm kernel shell biochar promotes root health and rhizosphere biodiversity in young oil palm seedlings infected with *Ganoderma boninense*

**DOI:** 10.3389/frmbi.2026.1742803

**Published:** 2026-02-13

**Authors:** Saran Anandan, Asgar Ali, Anurita Selvarajoo, Christina Vimala Supramaniam

**Affiliations:** 1 School of Biosciences, University of Nottingham Malaysia, Semenyih, Malaysia; 2 Department of Integrative Agriculture,College of Agriculture and Veterinary Medicine, United Arab Emirates University, Al-Ain, United Arab Emirates; 3 Department of Civil Engineering, University of Nottingham Malaysia, Semenyih, Malaysia; 4 School of Science, Faculty of Engineering and Science, University of Greenwich, Chatham, United Kingdom

**Keywords:** antagonistic fungi, metagenomics, oil palm, trichoderma, white rot fungi

## Abstract

Oil palm (*Elaeis guineensis*) contributes up to 3% of gross domestic product (GDP) in Malaysia. Long-term monoculture production reduced natural biodiversity and increased severe threat by *Ganoderma boninense*, a causal agent of basal stem rot (BSR) disease. BSR recorded projections of 860,610 hectares of plantations to be devastated by BSR by 2040. While disease management has prioritised good sanitation practices, *Trichoderma* spp. is a potential solution to combatting *G. boninense*. In this study, we determined the efficacy of *Trichoderma* spp. isolate 4A added to palm kernel shell (PKS) biochar (T-mix) to improve oil palm root health. Three-month-old seedlings were observed in control treatments, T1 to T4 and *Trichoderma* sp. treatments, T5 to 12 with Ganoderma added in T7,8, 11 and 12. Root development parameters such as root architecture, length, diameter, and surface area were observed every two months for six months. Root length of T5 (3.3 m) and T9 (4.4 m) was higher than no-treatment control, T1 (2.5 m) indicating *Trichoderma* sp. support of root health. T9 (T-mix) has significantly improved root architecture in root scan with denser and multiple root branches as while all other diseased oil palms exhibit stunted roots. The diameter of roots shows similar trend to root length of T9 roots with the highest reading at 5.4 mm. T11 showed the overall improved fungal biodiversity at 6 months post inoculation with potential disease suppressive effects against other common pathogens such as *Fusarium* sp. This study highlights a new perspective of *Trichoderma* spp. treatment with biochar to provide protection to growing young oil palm root health, beyond disease control, indicating a beneficial role for early application at seedling stage. For long term application, *Trichoderma* spp. combined with biochar support healthy fungal dynamics without over-dominating indigenous fungal inhabitants. This is the first study to highlight the role of combined *Trichoderma* spp. and biochar in influencing the root architecture and rhizosphere dynamics of a perennial oil palm at the seedling stage. Overall, this study presents an exciting opportunity to use a new *Trichoderma* sp.-biochar solution in the battle against *G. boninense*.

## 1 Introduction

Oil palm is the most productive major oil crop, supplying over one-third of global vegetable oil while occupying a relatively small proportion of agricultural land (Barcelos et al., 2015; Mayes, 2020). In Malaysia, palm oil contributes substantially to agricultural output and national gross domestic product (Chang, 2021). However, the long-term reliance on monoculture has increased vulnerability to specialised pests and pathogens, with basal stem rot (BSR) caused by *Ganoderma boninense* now recognised as the most destructive disease of oil palm (Wong et al., 2012; McDonald and Stukenbrock, 2016). BSR is estimated to cost the Malaysian industry up to RM1.7 billion annually, with projections suggesting extensive future plantation losses if effective management strategies are not implemented (Seman, 2018; Olaniyi and Szulczyk, 2020). *Ganoderma boninense* initially behaves as a biotrophic pathogen, secreting cell wall-degrading enzymes to colonise host tissue with limited visible symptoms, before switching to a necrotrophic phase that degrades xylem and severely disrupts water transport, ultimately killing the palm (Bahari et al., 2018; Goh et al., 2014, 2018).Current BSR management relies mainly on sanitation practices such as removal of infected material and attempts to reduce inoculum in soil, with supplementary use of fungicides and biopesticides (Maluin et al., 2019; Siddiquee et al., 2009; Puspita et al., 2023). Among biocontrol agents, *Trichoderma* spp. have attracted sustained interest because of their broad antagonistic activity and ability to colonise the rhizosphere of diverse crops. *Trichoderma* spp. have long been recognised as effective antagonists of soil-borne pathogens through mechanisms including mycoparasitism, antibiosis and competition (Weindling, 1932; Mihuta-Grimm and Rowe, 1986; Yedidia et al., 1999). They also act as plant growth-promoting fungi, enhancing root development and biomass by modulating hormone signalling, particularly auxin-related pathways, and by improving nutrient availability (Contreras-Cornejo et al., 2009; Brotman et al., 2013). In oil palm and related pathosystems, *Trichoderma* spp. have been shown to suppress *Ganoderma* spp. via secretion of cell wall-degrading enzymes, production of antifungal volatile organic compounds and mycoparasitism, while simultaneously improving seedling growth (Widyastuti, 2006; Muhd Zainuddin and Abdullah, 2008; Siddiquee et al., 2009; Muniroh et al., 2019). Biochar, a porous carbon-rich material produced through thermochemical conversion of biomass in an oxygen-limited environment, is increasingly used as a soil amendment to improve fertility, moisture retention and cation exchange capacity (International Biochar Initiative, 2015; DeLuca et al., 2015; Rawat et al., 2019). Plant-derived biochars, including those produced from agricultural residues, are particularly attractive for large-scale application due to their relative cost-effectiveness compared with animal-waste-derived biochars (Sanford et al., 2022). Beyond their physico-chemical benefits, biochars can serve as carriers and protective habitats for beneficial microorganisms because of their high surface area and sorption capacity (Ajeng et al., 2020). Several studies have demonstrated that biochar-enriched formulations of biocontrol agents such as *Aspergillus*, *Bacillus*, *Streptomyces*, *Pseudomonas* and *Trichoderma* can enhance disease suppression and plant health in a range of crops, including reductions in *Phytophthora capsici* disease in pepper, control of Fusarium wilts in tomato and mitigation of viral and viroid infections in tomato (Wang et al., 2019; Hasan et al., 2020; Luigi et al., 2022; Da Silva et al., 2022).Despite recent advances in the biological control of basal stem rot (BSR), the effects of *Trichoderma* spp.–biochar combination on both root system architecture and rhizosphere microbial diversity in woody perennial crops such as oil palm remain poorly characterised. Existing work on BSR biocontrol in oil palm has largely focused on direct antagonism of *Ganoderma boninense* by *Trichoderma* spp. in seedlings or mature palms, without a biochar component or detailed analysis of root traits or rhizosphere community structure (Paterson, 2019). At the same time, a substantial literature has developed around biochar as a soil amendment that improves soil fertility, modifies microbial communities and can reduce plant disease severity, but usually without *Trichoderma* spp (Elad et al., 2010; Biederman & Harpole, 2012; Palansooriya et al., 2019). Only a small number of studies have begun to integrate *Trichoderma* spp. with biochar for disease management, either in oil palm or other crops, and these typically emphasise disease incidence and bulk growth responses rather than linking specific root traits with high-resolution rhizosphere microbiome data (de Medeiros et al., 2021; Yao et al., 2023; Ahmad et al., 2024; Paveen et al., 2025). To our knowledge, no previous work has simultaneously quantified fine-scale root system architecture and rhizosphere microbial diversity in *Ganoderma* spp.-infected versus non-infected oil palm seedlings treated with *Trichoderma* spp.–biochar formulations.

The present study addresses this gap by evaluating the efficacy of a combined *Trichoderma asperellum* 4A and oil palm kernel shell (PKS) biochar formulation, hereafter referred to as T-mix, in promoting root health and enhancing rhizosphere microbial diversity in young oil palms. We compared T-mix with a *Trichoderma* sp. conidial suspension applied alone, under both standard and additional organic fertiliser regimes, in seedlings that are either previously infected or uninfected with *Ganoderma* spp. Root development is characterised using total root length, average root diameter, total root surface area and root architecture quantified with the WinRhizo imaging system, while rhizosphere microbial diversity is assessed to determine community-level responses to the treatments. By coupling detailed root phenotyping with rhizosphere microbiome profiling, this work provides new insight into the potential of a *Trichoderma* spp.–biochar combination to foster disease-suppressive conditions in oil palm nurseries and offers a mechanistic basis for integrating such formulations into BSR management strategies.

## 2 Materials and methods

### 2.1 Source of oil palm, biochar and biofertiliser

Young oil palms (AA Hybrida, Applied Agricultural Resources SB, Malaysia) were obtained as 3-month-old rooted ramets and were grown and maintained in a shade house. The bio-fertiliser (commercially known as living organic fertiliser (LOF)) was derived from an industrial composting process of empty fruit bunch (EFB), decanter cake, palm oil mill effluent (POME) and boiler ash while flaky palm kernel shell biochar was a by-product from the extraction of crude palm oil extraction. Both products were obtained from Greenplant Organics Sdn Bhd, Palong, Malaysia. All research activities in this study were carried out at the University of Nottingham Malaysia, Semenyih, Malaysia.

### 2.2 *Trichoderma* sp. source

*Trichoderma asperellum* 4A (Genbank accession number: KM456217.1) was originally isolated from Balau Estate, Semenyih, Malaysia and was maintained fortnightly in potato dextrose agar (PDA) (Oxoid).

### 2.3 Preparation of *Trichoderma asperellum*-biochar mixture

*Trichoderma asperellum* 4A was grown in PDA supplied with 250 µg/l of streptomycin (HiMedia) and 250 µg/l of chloramphenicol (Nacalai Tesque) for 14 days (Gil et al., 2009). Cultures were dislodged from standard 9 mm Petri dishes using autoclaved distilled water before being gently scraped with a glass microscope slide (Musa et al., 2018). The conidial cultures were diluted in sterile malt extract broth (MEB) (Oxoid) at 1:10 dilution and allowed to grow at room temperature, overnight in an orbital shaker at 100 rpm. The liquid cultures was adjusted to a concentration of 10^6^ spore/ml using MEB and were applied directly to oil palm pots. For T-mix, the same concentration of liquid cultures was mixed with PKS biochar at 100% w/v. T-mix was stored in high density polyethylene (HDPE) bags for up to six months at room temperature.

### 2.4 *Ganoderma* sp. source and preparation

**Figure 1:**
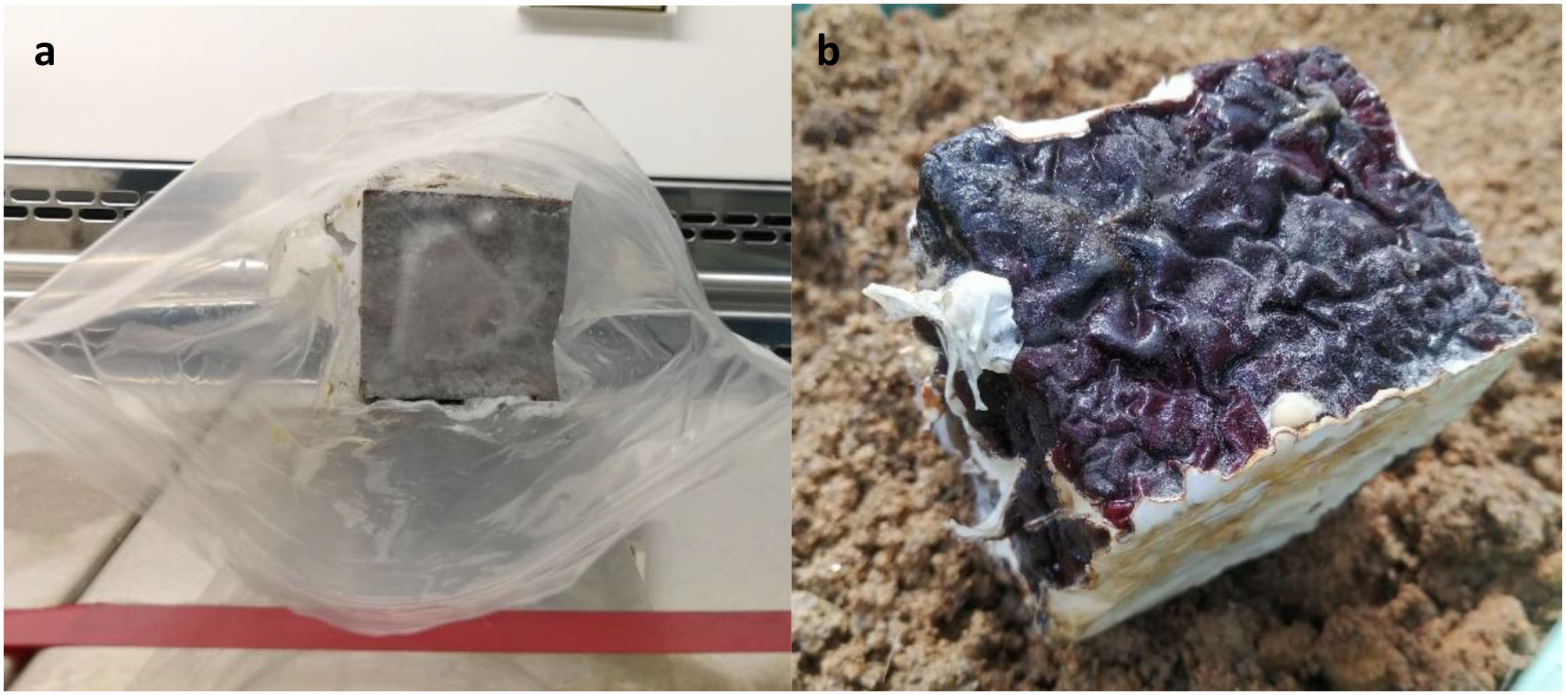
**(a)** *Ganoderma* sp. inoculated on RWB; **(b)** Mature *Ganoderma* on RWB, with dark-red fruiting bodies.

*Ganoderma boninsense* GBLS was obtained from Lian Seng Estate, Malaysia and characterised from a previous study (Goh et al., 2018). *Ganoderma boninsense* cultures was maintained in PDA. Two-week-old GBLS cultures grown in 9 mm Petri dish with PDA were inoculated on untreated 6cm × 6cm × 6cm rubber wood blocks (RWBs) (Rubber Research Institute Malaysia) by adding mycelia from one Petri dish per RWB. The RWBs were then placed in zip lock bags and stored away from sunlight to grow for six weeks (**Figure 1**) (Sariah et al., 1994).

### 2.5 Experimental design

Plant inoculation experiment was carried out with 180 young oil palms arranged in a complete block design. Each of the four blocks of treatments were given three levels of biological control with 15 palms for each level (**Table 1**). The fertiliser treatments were:

**Table 1:** Experimental design with treatments on three-month old, rooted oil palm ramets.

Treatments	Standard fertiliser (healthy control)	Standard fertiliser + LOF (organic treatment)	Standard fertiliser + *G. boninsense* RWB (disease control)	Standard fertiliser + LOF + *G. boninsense* RWB (disease treatment)
No treatment control	T1	T2	T3	T4
*T. asperellum* only	T5	T6	T7	T8
T-mix	T9	T10	T11	T12

1. standard fertiliser – control, healthy
2. standard fertiliser with LOF bio-fertiliser – treatment control, healthy
3. standard fertiliser – diseased
4. standard fertiliser with LOF bio-fertiliser – diseased

The three levels of biological control were:

T1-T4: no treatment control
T5-T8: *Trichoderma asperellum* 4A
T9-T12: T-mix: *Trichoderma asperellum* 4A + PKS biochar

### 2.6 Infection assay

Assay was conducted according to (Izzati and Abdullah, 2008) with slight modification. Three-month old oil palm plants were transplanted from their germination tray pots into polyethylene planting bags measuring 25cm × 25cm and allowed to acclimatise for one month before treatments were delivered.

The soil was prepared by mixing topsoil and river sand at a ratio of 1:1, and each planting bag contained approximately 4 kg of soil. LOF was applied according to manufacturer’s recommendation by mixing with soil in treatments T2, T4, T6, T8, T10 and T12. The plants were watered every day for 30 minutes using a garden hose with a spray nozzle. Upon acclimatisation, plants in diseased treatments (T3, T4, T7, T8, T11 and T12) were transplanted into new planting bags containing the same soil mix as before but with added RWBs inoculated with *G. boninense*. One week later, 100g of T-mix was applied on the surface of the soil for T9-T12 and for treatments with *T. asperellum* 4A only, T5-T8, 100 ml of liquid conidial suspension was delivered directly to the soil surface.

All plants received grower standard practice of inorganic fertiliser such as Bayfolan^®^ foliar fertiliser for three months and subsequent rock phosphate and NPK fertiliser as recommended by AARSB, Malaysia.

The plants were destructively sampled at the 2^nd^, 4^th,^ and 6^th^ month post treatment. Root samples were obtained at every time point and root development parameters (total root surface area, total root length, average root diameter) were analysed using WinRhizo 2013e (Regent Instruments).

### 2.7 DNA sequencing of rhizosphere soil samples

Rhizosphere DNA was collected from soil attached to roots at 6 months post inoculation. DNA wasextracted using DNA Powersoil Pro kit (Qiagen) according to manufacturer’s recommendation.The ITS1 region was amplified from gDNA using the primer pair BITS ACCTGCGGARGGATCA and B58S3 GAGATCCRTTGYTRAAAGTT (Klindworth et al., 2013; García-López et al., 2020). An additional 5 bases of inline barcode and partial Illumina adapter were incorporated at the 5’ end of the primers to enable inline barcoding (Glenn et al., 2019). Different samples were amplified using different combinations of the forward and reverse inline primers. PCR was performed using Rediant II PCR mastermix (Apical Scientific, Malaysia) using the PCR profile of: 95C for 2 minutes followed by 30 cycles of 95C for 15 s, 50C for 30s and 72C for 30s. The barcoded amplicons were subsequently visualised on gel and purified using 0.8 X vol. of SPRI bead. The purified amplicons were used as the template for 8 cycles of index PCR to incorporate the complete Illumina adapter and Illumina-compatible dual-index barcodes.

The constructed libraries were subsequently size-selected using 0.8 X vol of SPRI bead and pooled into a single tube. Quantification of the pooled libraries used Denovix high sensitivity assay. Sequencing of the pooled libraries was performed on a NovaSEQ6000 (Illumina, San Diego) using the 2x150bp paired-end sequencing configuration.

Demultiplexing and primer trimming of the raw paired-end reads used cutadapt v1.18 (Martin, 2011). The trimmed reads were subsequently merged usingfastp v0.21 (Chen et al., 2018). The processed readswere imported into QIIME2 v.2023.9 (Bolyen et al., 2019) and denoised into Amplicon Sequence Variant (ASV) with dada2 v.1.26.0 (Callahan et al., 2016). Taxonomic assignment of the ASV used q2-feature-classifier (Bokulich et al., 2018) that has been trained on the latest Unite V9 (McDonald et al., 2023).

ASVs (amplicon sequence variants) with taxonomic assignment to at least the phylum level were selected for subsequent analysis. Following data rarefaction to the sample with the lowest number of reads, alpha diversity analysis was conducted in QIIME2. Diversity indices including Chao1, ACE, Shannon, Simpson, and Faith_PD were calculated to assess within-sample diversity. For beta diversity analysis, distances based on Jaccard, Bray Curtis, weighted UniFrac, and unweighted UniFrac were computed using the rarefied dataset.

### 2.8 Statistical analyses

The results obtained were analysed using IBM SPSS Statistics version 28. For *Trichoderma* viability experiment, independent samples T-test was used to analyse the data to identify any significant difference between the two concentrations of *Trichoderma*. The root development parameter results were analysed using tests of between-subjects effects, and Tukey’s multiple comparisons test was carried to analyse the difference of means between treatments.

## 3 Results and discussion

### 3.1 Effect of treatment on total cumulative root length of young oil palm

**Figure 2:**
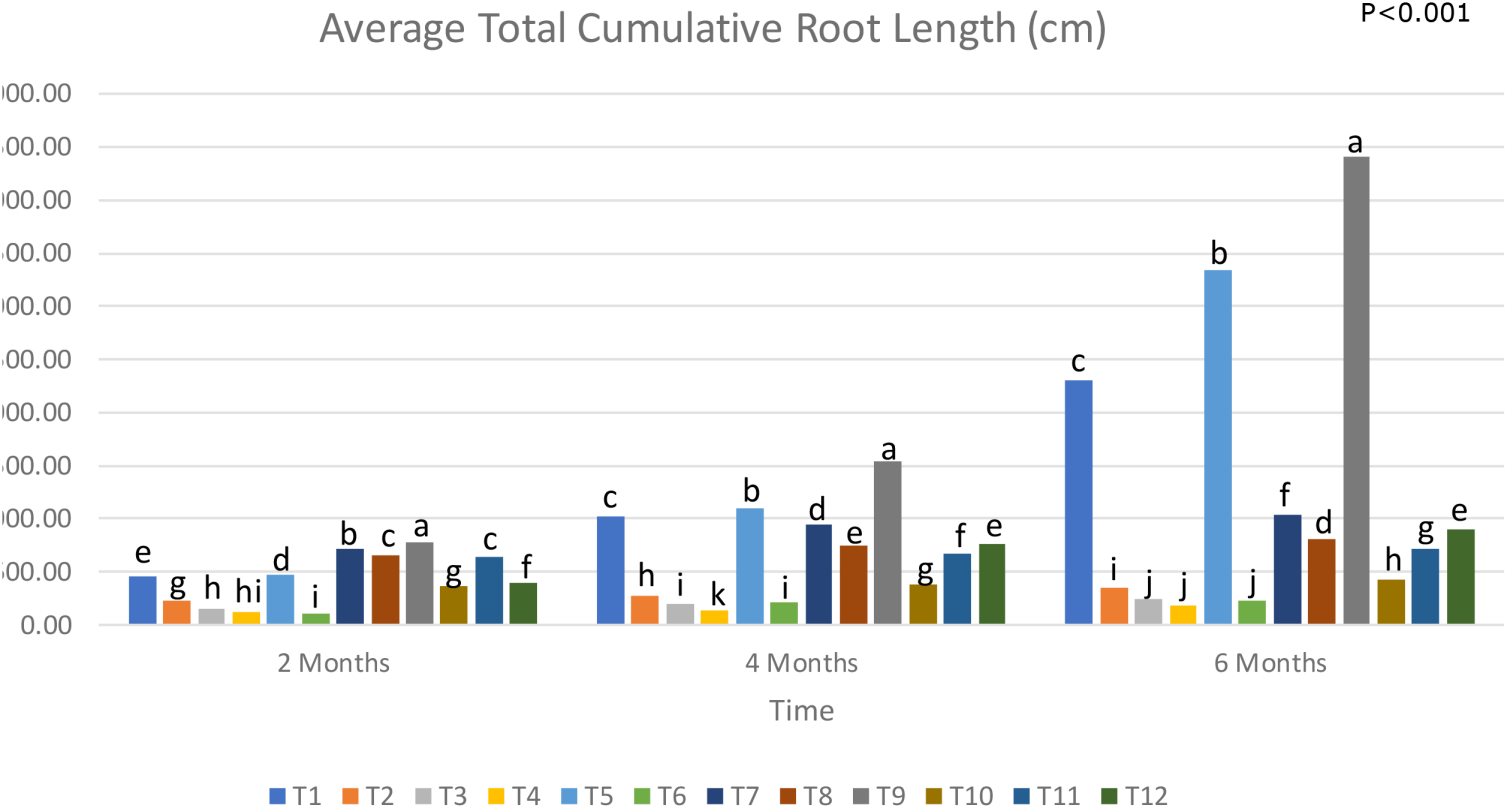
Total root length of oil palm seedlings under treatments from T1 to T12 analysed from 2 to 6 months (T1, Absolute control (no LOF, no biological control); T2, LOF only; T3, *G. boninense* only; T4, *G. boninense* + LOF; T5, *T. asperellum* only; T6, *T. asperellum* + LOF; T7, *T. asperellum* + *G. boninense*; T8, *T. asperellum* + *G. boninense* + LOF; T9, T-mix only; T10, T-mix + LOF; T11, T-mix + *G. boninense*; T12, T-mix + *G. boninense* + LOF). Plots with different alphabetic letters were significantly different at P<0.001 using Tukey’s Multiple Comparisons Test.

**Figure 3:**
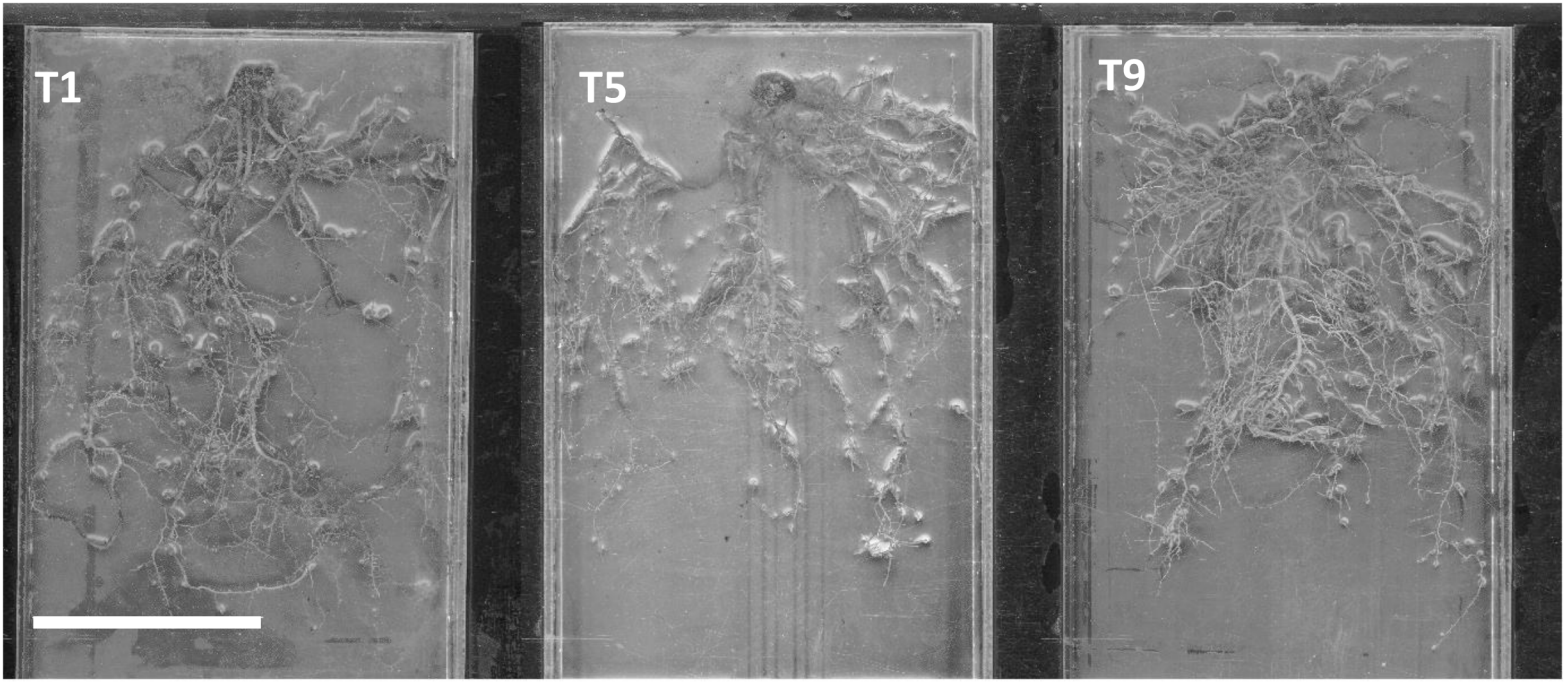
Root scan images of young oil palm roots (T1, absolute control; T5, *T. asperellum* only; T9, T-mix only). Scale bar=10 cm.

Overall, T5 and T9 displayed the highest results, with T5 having an almost 45% increase in total root length more than T1, and T9 having almost double the total root length of T1 after 6 months (**Figure 2**). This indicates the ability of *Trichoderma* spp. to induce root growth in young oil palms. In a related study, *T. harzianum-*induced total root length in tomato plants is regulated by the *qid74* gene, which is shown to not only increase the length of lateral roots and secondary root hairs but increased the total absorptive surface of the roots and thus allowing for an increased uptake in nutrients (Samolski et al., 2012). T9 performed better than T5, suggesting that a mixture of *Trichoderma* spp. and biochar performs better than an application of pure *Trichoderma* spp. in boosting total root length (**Figure 3**). This is evidenced by findings by Hasan et al. in 2020 which showed that a mixture of *T. asperellum* and coconut fibres performed better than an application of pure *Trichoderma* alone.

In retrospective, healthy treatments that were supplied with LOF (T2, T6 and T10), recorded relatively low total root lengths. Addition of organic carbon may increase the overall required C/N ratio, affecting root growth (Nie et al., 2013). However, T10 had a significantly higher total root length than T6, which suggests that a combination of *Trichoderma* spp. with biochar was more effective at mitigating the inhibitory effects of LOF when compared to a liquid *Trichoderma* spp. application. This hypothesis can be further supported by comparing the total root length between treatments 8 and 12. The combination of *Trichoderma* spp. with biochar produced a higher total root length in diseased plants than a liquid *Trichoderma* spp. application. Treatments 3, 4, and 6 had no significant difference in root length, suggesting that a pure *Trichoderma* spp. application in tandem with LOF on healthy plants yielded the same results as diseased plants. The lower root lengths observed in treatments that received LOF suggest that in this study, LOF may have negatively affected root growth. There is evidence to show that organic fertilisers may contain trace levels of heavy metals (Wang et al., 2013). Heavy metals such as chromium have been shown to depress root elongation despite supplementation with nitrates (Panda and Patra, 2000). Although, in this study, no trace metal was detected in LOF, batch to batch variation could occur in the production facility.

**Figure 4:**
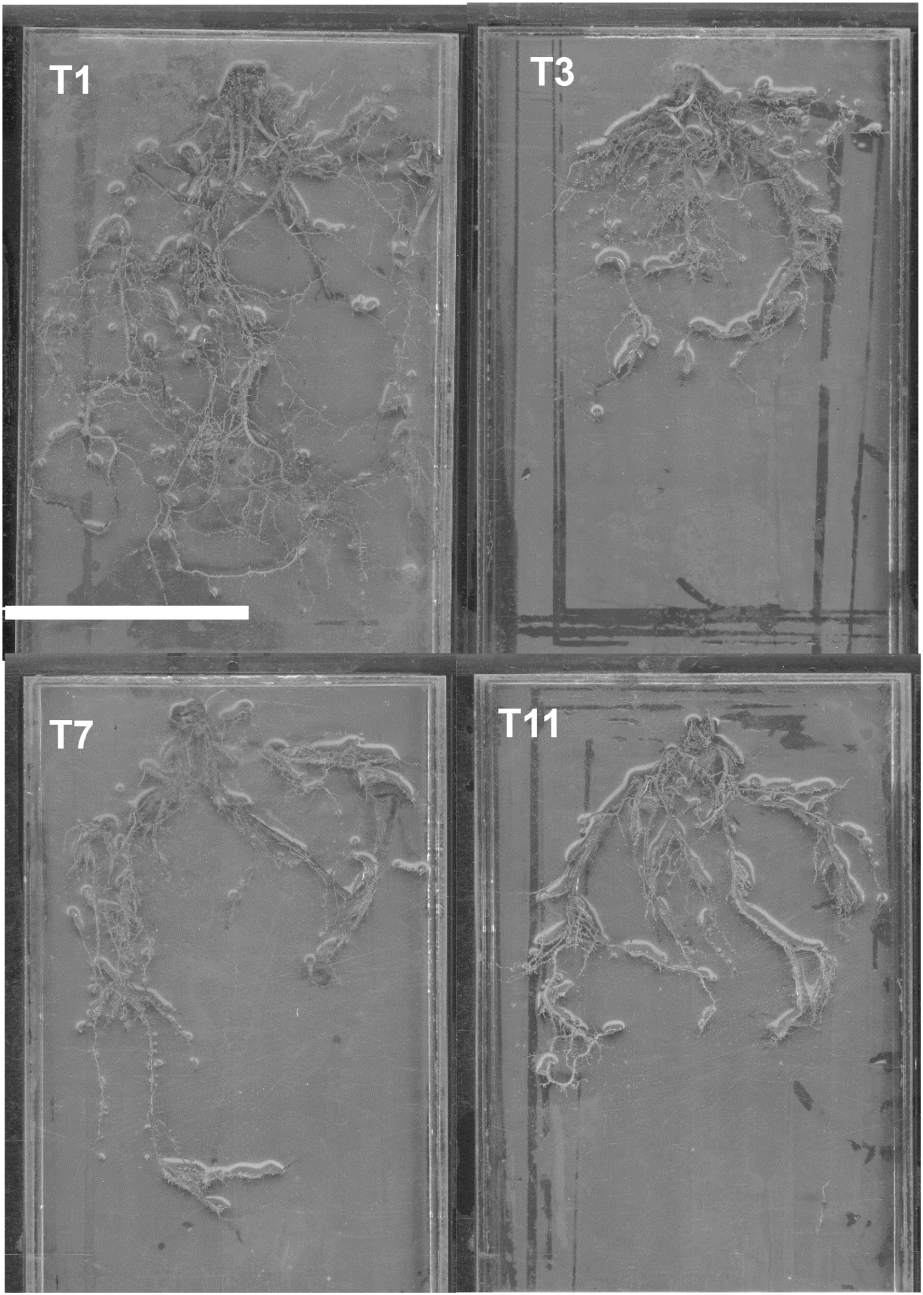
Root scan images of young oil palms infected with *Ganoderma boninense* compared to absolute control. (T1, absolute control; T3, *G. boninense* only; T7, *T. asperellum* & *G. boninense* only; T11, T-mix & *G. boninense* only). Scale bar=10 cm.

The diseased treatments all recorded significantly lower total root length than the healthy plants that did not receive LOF application (**Figure 4**). *Ganoderma* spp. infection led to necrosis and decay of oil palm roots, which will in turn reduce total root length. The secretion of CWDEs not only reduces root surface area, but root length as well, as the action of enzymes such as cellulases, pectinases and polygalacturonases result in the destruction of root cell walls (Ghasemi et al., 2020; Zhang et al., 2022; Rees et al., 2009; Gawade et al., 2017). The degradation of root cell walls in turn causes the root cells to weaken and collapse, thus resulting in a reduced total root length. *Ganoderma* spp. is also shown to affect the hormonal balance in oil palms. Oil palms infected with *Ganoderma* spp. have been shown to display an upregulation of auxin regulators, which in turn limit auxin production and signalling (Bahari et al., 2018). As auxin plays a role in root growth and elongation, a disruption in auxin signalling results in reduced total root length. Another negative effect of *Ganoderma* spp. on oil palm roots is the induction of oxidative stresses. Bahari et al. observed the upregulation of reactive oxygen species (ROS) elicitors in oil palms infected with *Ganoderma* spp. A common plant defense mechanism is the promotion of ROS production through an oxidative burst. As early colonisation of *Ganoderma* spp. takes place in the roots, it is possible that the high levels of ROS produced during an oxidative burst may inhibit root growth due to DNA damage in the root elongation zone (Tsukagoshi, 2016).

Diseased plants treated with liquid *Trichoderma* spp. (T7) recorded a higher total root length than the diseased treatment that had both *Trichoderma* spp. and LOF (T8). This is similar to the difference observed between Treatments 11 and 12, in which the diseased treatment with T-mix and LOF performed better than the diseased treatment with only T-mix. This further strengthens the suggestion that T-mix was more effective at controlling the possible harmful effects of LOF, as improvements can be seen even in diseased young oil palms. However, an application of pure *Trichoderma* spp. was shown to be more effective than T-mix at increasing the total root length of diseased oil palms that did not receive LOF.

### 3.2 Effect of treatment on average root diameter

**Figure 5:**
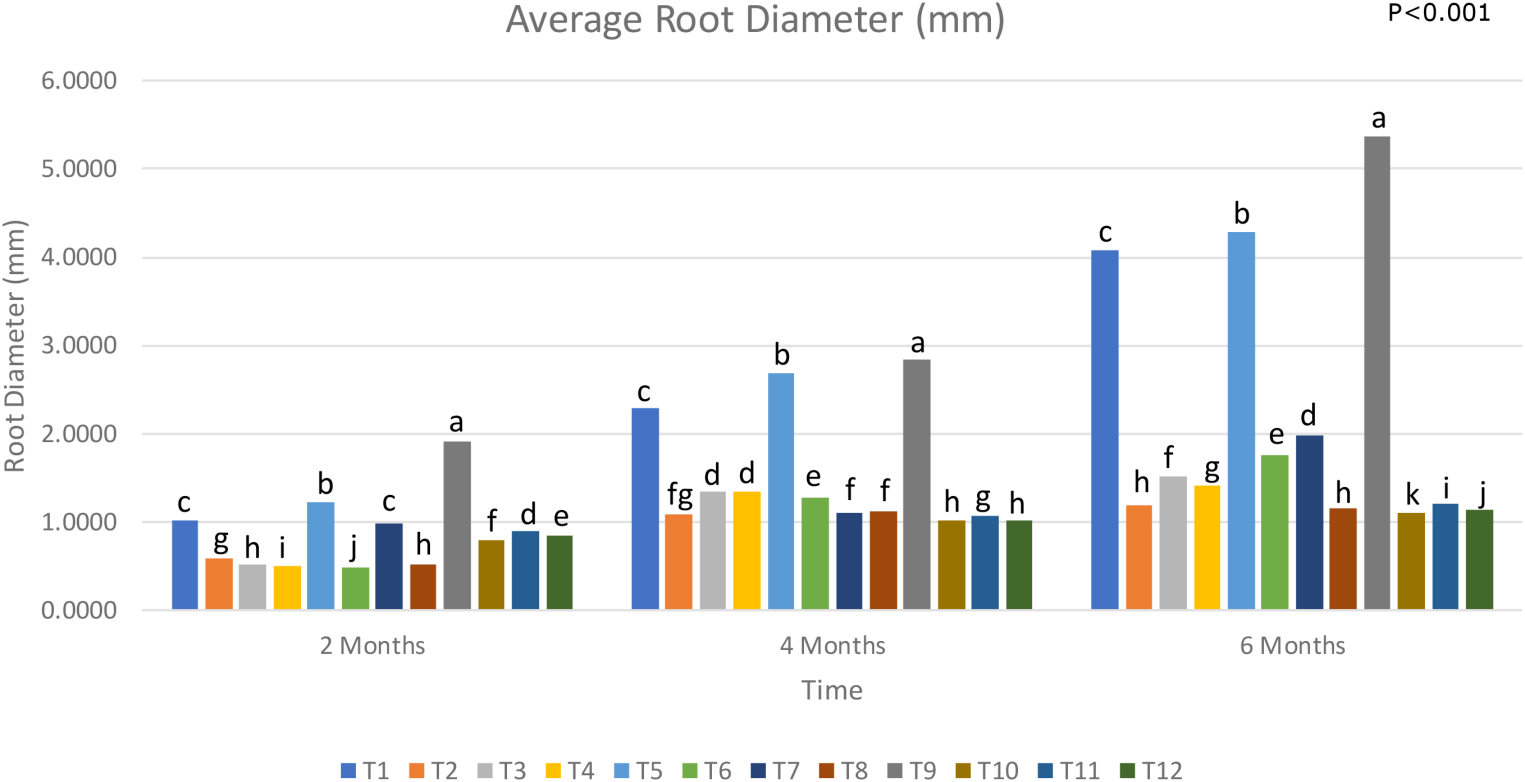
Average root diameter of oil palm seedlings under treatments from T1 to T12 analysed from 2 to 6 months(T1, Absolute control (no LOF, no biological control); T2, LOF only; T3, *G. boninense* only; T4, *G. boninense* + LOF; T5, *T. asperellum* only; T6, *T. asperellum* + LOF; T7, *T. asperellum* + *G. boninense*; T8, *T. asperellum* + *G. boninense* + LOF; T9, T-mix only; T10, T-mix + LOF; T11, T-mix + *G. boninense*; T12, T-mix + *G. boninense* + LOF). Plots with different alphabetic letters were significantly different at P<0.001 using Tukey’s Multiple Comparisons Test.

All the non-LOF treatments (Treatments 1, 3, 5, 7, 9 and 11) recorded a higher average root diameter than their counterparts that received LOF (Treatments, 2, 4, 6, 8, 10 and 12) (**Figure 5**). In following with the trend observed in the previous root growth parameters, it can be established that LOF is not beneficial to the root development of young oil palms, regardless of plant health. For young oil palm requiring high amounts of nitrogen for growth, reduced organic input is suggested. This is the opposite for mature palms within a plantation, where the organic input is necessary to maintain long term soil health (Sundram et al., 2019; Pauli et al., 2014);.

T5 and T9 had the highest average root diameter, which was consistent with the findings of the previous root development parameters. While the difference between Treatment 5 and the absolute control was significant, the difference was not as stark as that between Treatment 9 and the absolute control. Treatment 9 had a 31% increase in average root diameter when compared to Treatment 1, and a 25% increase when compared to Treatment 5. The application of *Trichoderma* spp.-biochar combination was more effective at increasing the average root diameter in healthy young oil palms than liquid *Trichoderma* spp. alone. To the author’s knowledge this is the first study to determine the effect of *Trichoderma* spp. application on the root health in young oil palm in combination with palm biowaste such as biochar. In a related study, Sani et al., in 2020 reported that a combination of *Trichoderma* spp. and biochar significantly boosted the health and root growth of tomato plants when compared to an application of *Trichoderma* spp. alone.

The oil palms seedlings that were infected with *Ganoderma* spp. all displayed significantly lower average root diameter when compared to healthy oil palms without LOF. *Ganoderma* spp. has been shown to affect the hormonal balance of oil palms. According to Bahari et al., a *Ganoderma* spp. attack can result in the downregulation of ethylene and gibberellin signalling pathways. As ethylene and gibberellin play an important role in root radial growth, a downregulation would result in a reduced root diameter (Tadeo et al., 1997). The accumulation of ROS in root cell walls due to oxidative bursts can also result in cell wall degradation, and thus a reduction in average root diameter. On a larger scale, the widespread damage caused by *Ganoderma* spp. on the collective root architecture of oil palms will reduce the average root diameter, leading to higher disease incidence and palm death in a plantation.

### 3.3 Effect of treatment on total root surface area

**Figure 6:**
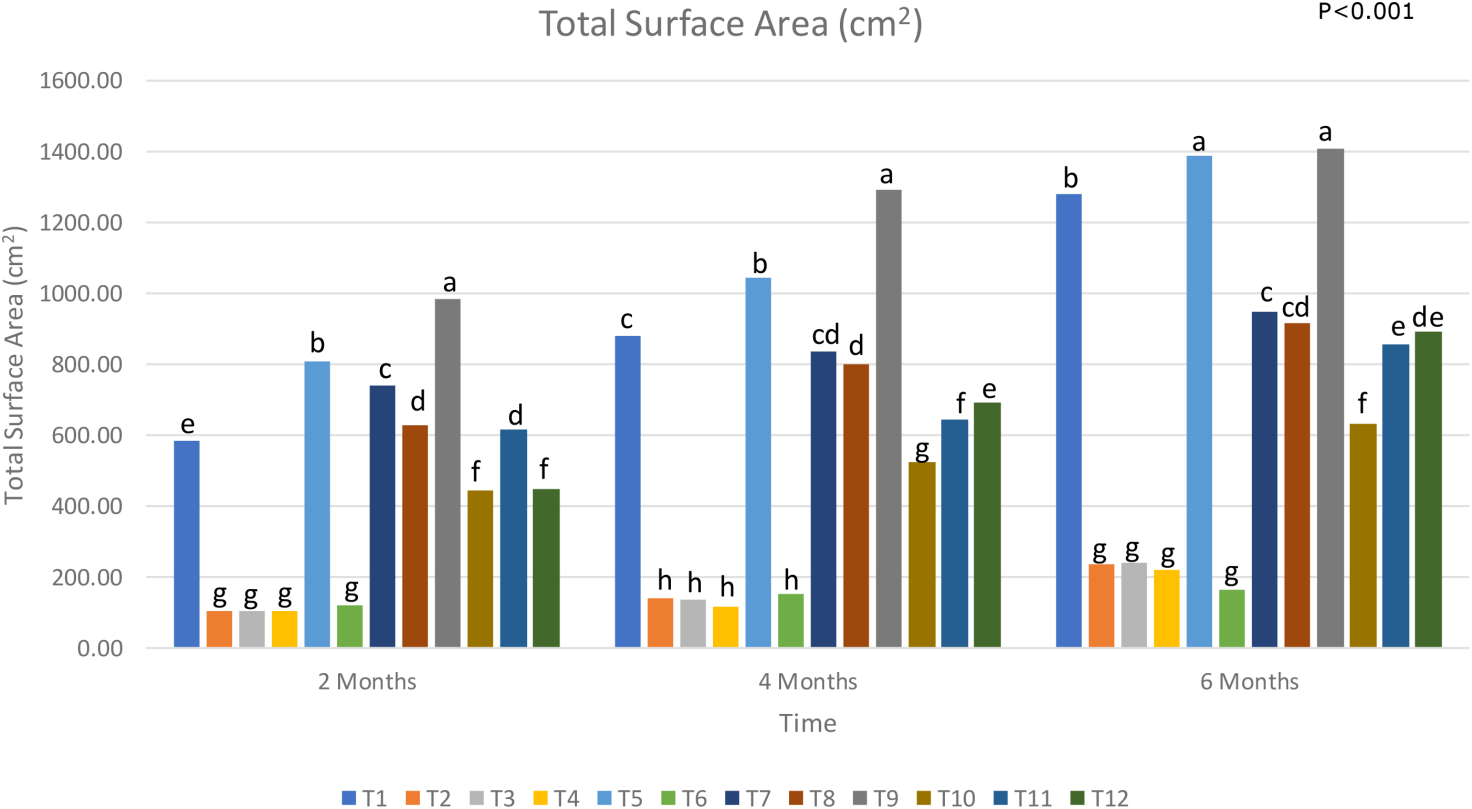
Total root surface area of oil palm seedlings under treatments from T1 to T12 analysed from 2 to 6 months (T1, Absolute control (no LOF, no biological control); T2, LOF only; T3, *G. boninense* only; T4, *G. boninense* + LOF; T5, *T. asperellum* only; T6, *T. asperellum* + LOF; T7, *T. asperellum* + *G. boninense*; T8, *T. asperellum* + *G. boninense* + LOF; T9, T-mix only; T10, T-mix + LOF; T11, T-mix + *G. boninense*; T12, T-mix + *G. boninense* + LOF). Plots with different alphabetic letters were significantly different at P<0.001 using Tukey’s Multiple Comparisons Test.

T5 and T9, which contain *Trichoderma* spp., consistently recorded the highest total root surface area, suggesting that *Trichoderma* spp. aids in increasing root surface area (**Figure 6**). Meng et al., 2019 reported the function of a *T. guizhouense* protein, *Tg*SWO, that behaved similarly to plant expansion proteins and played a promoted the growth of cucumber roots. *Trichoderma viride* was shown to increase the total root surface area in liquorice plants (He et al., 2020). A study by Yedidia et al. highlighted the positive effect that *T. harzianum* had on the root surface area of cucumbers. The significant differences between the two treatments in the 2^nd^ and 4^th^ month of sampling also suggest that *Trichoderma* spp. may boost root surface area when used in tandem with a waste resource such as biochar, as evidenced by Vecstaudza et al. in 2018. However, the final month of sampling showed no significant differences in total root surface area between T5 and T9. Further studies of longer than six months may be required to determine the efficacy between applying liquid *Trichoderma* spp. and solid *Trichoderma* spp. mixed in biochar.

T2, T4 and T6, with LOF, displayed similar levels of root surface area growth as T3, the Ganoderma disease control. T2 and T6, despite not being infected with *Ganoderma* spp., showed consistently low total root surface area throughout the six months. Parallels between the low total root surface area can be seen across all three levels of biological control in the healthy treatments supplied with LOF (Treatments 2, 6 and 10) in comparison with the healthy treatments that were only supplied with chemical fertiliser, as evidenced in Treatments 6 and 10 in comparison to Treatments 5 and 9 respectively. The absence of any significant differences between Treatments 2, 3, 4 and 6 suggest that LOF did not support root surface area in young oil palms.

The treatments that were infected with *Ganoderma* spp. all displayed significantly lower total surface area than the healthy treatments without LOF. The difference between the total surface area of the diseased treatments and the healthy treatments were also shown to increase over the duration of six months. *Ganoderma* spp. primarily colonises oil palm through the roots, hence the extent of the damage to the roots is highly visible. Through the secretion of cell wall degrading enzymes (CWDEs), *Ganoderma* spp. can penetrate deeper and further damage root cell walls of oil palms (Rees et al., 2009). Alexander et al., in 2017 were able to show through scanning electron microscopy the effects of *Ganoderma* spp. on eight-month-old oil palm seedlings, in which *Ganoderma* spp. negatively modifies the root architecture of infected seedlings, resulting in a collapsed root cortex and disintegrated root epidermal and cortical cells. This results in a severely diminished total root surface area.

Diseased plants which received *Trichoderma* spp. and T-mix (T7 and T11), showed significantly higher total root surface area compared to untreated disease control (T3). The total root surface area recorded in the 6^th^ month was almost four times higher in Treatments 7 and 11 in comparison with Treatment 3. This large difference in results proves that *Trichoderma* spp. is highly capable at suppressing the effects of *Ganoderma* spp. *Trichoderma* spp. is shown to suppress the activity of *Ganoderma* spp. through the inducing of peroxidase and polypherol oxidase in oil palm seedlings, as well as boost plant growth (Musa et al., 2018). Muniroh et al., in 2019 showed that *T. asperellum* boosted plant growth through the production of indole acetic acid (IAA), a plant growth promoter, as well as phosphate solubilisation and siderophore production, both of which allowed for easier access to nutrients for plants. By comparing these findings with the total root surface area data observed by Treatments 7 and 11, it can be surmised that *Trichoderma* spp. and T-mix is successful at both controlling *Ganoderma* spp. and boosting the root development of young oil palms.

Diseased treatments that received LOF and a biological control (T8 and T12) had a higher total root surface area when compared to the healthy plants with a biological control that were supplied with LOF (T6 and T10). T8 and T12 also had a total root surface area that was about 4 times higher than T4, which was the diseased treatment that did not receive any biological control. This highlights the ability of *Trichoderma* spp. in improving the total root surface area of diseased young oil palms. However, the absence of significant differences between Treatments 7 and 8, and between Treatments 11 and 12 suggest that while the addition of *Trichoderma* spp. helps negate the inhibitory effects of LOF on the total root surface area, this does not improve the total root surface area of diseased young oil palms in comparison with just the addition of *Trichoderma* spp. alone.

## 4 Fungal biodiversity dynamics is influenced by Trichoderma and biochar treatments

**Figure 7:**
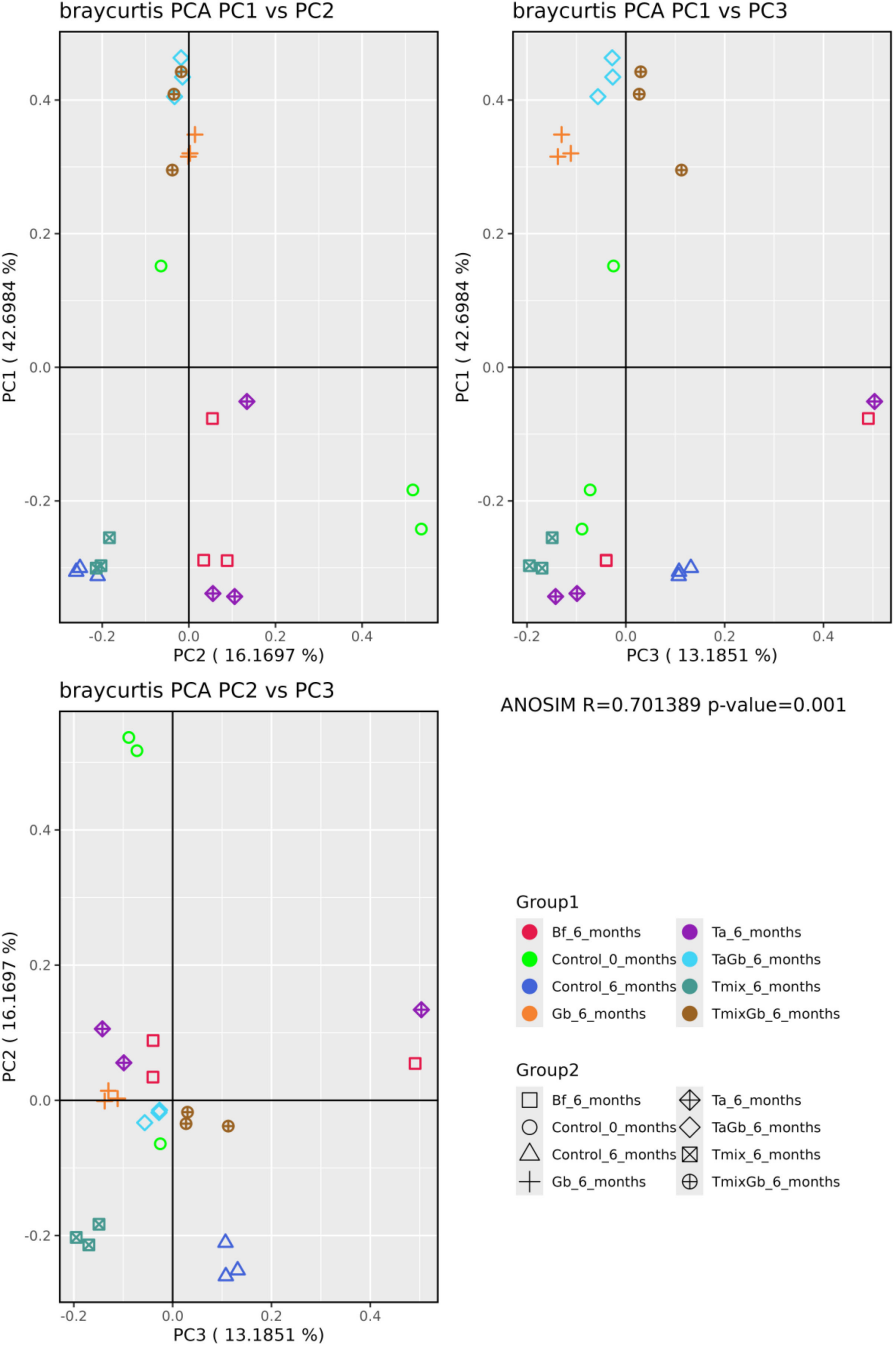
Beta diversity as assessed through Principal Coordinate Analysis (PCoA) plots representing three distinct dimensions: PC1 *vs*. PC2, PC1 *vs*. PC3, and PC2 *vs*. PC3. Distances between samples were determined using the Bray-Curtis metric, capturing compositional differences in microbial communities. In each PCoA plot, every point represents an individual sample, with colour and shape indicating the sample’s group classification as per the legend. Samples clustering closely together on the plots are indicative of shared microbial compositions, while those situated farther apart reflect greater dissimilarity in community structure.

**Figure 8:**
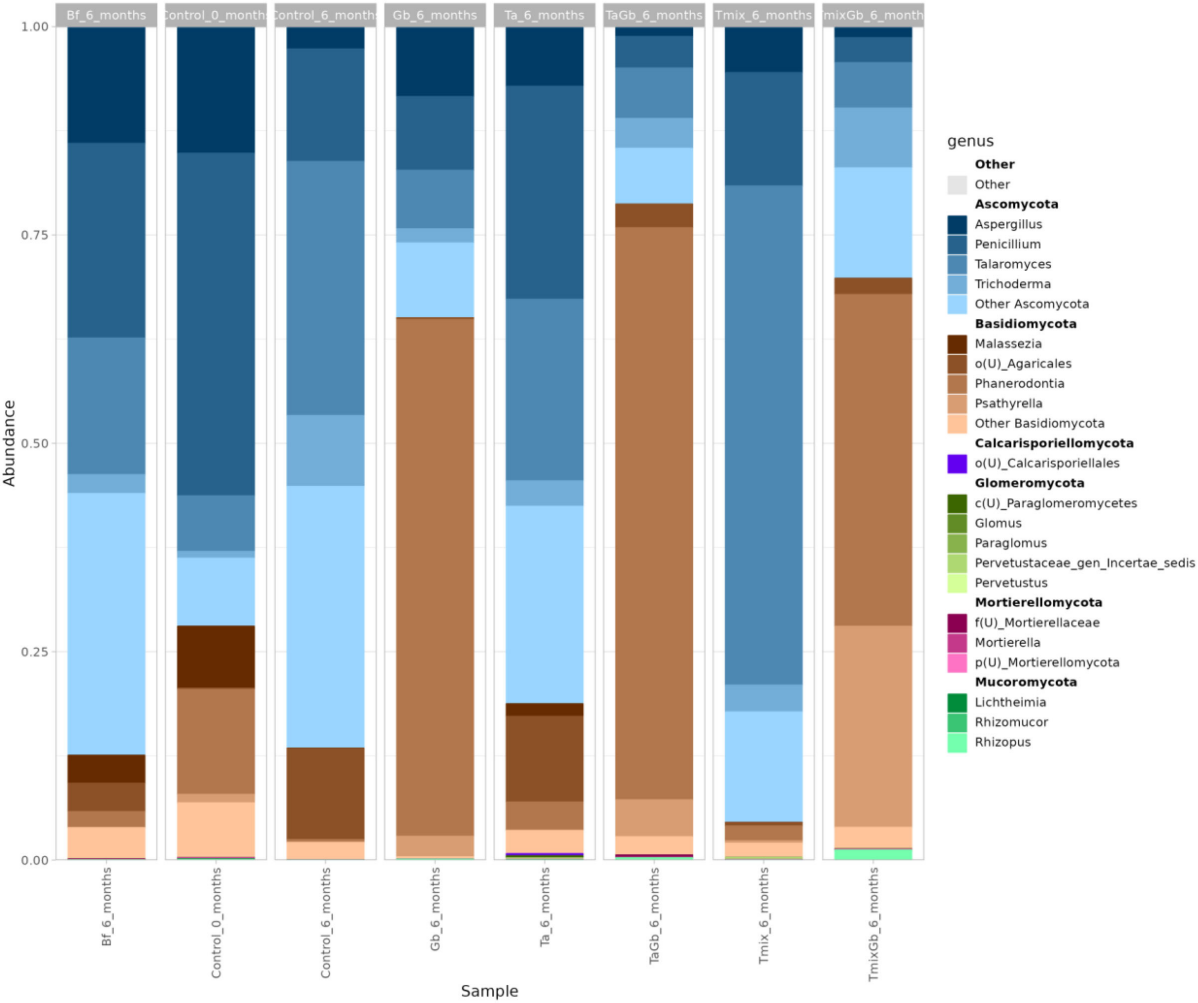
Stacked bar plot showing the average relative abundance of fungal genera per group.

The fungal diversity of soil samples across seven treatments was evaluated based on average Amplicon Sequence Variants (ASVs), Chao1 richness estimates, and Shannon diversity indices (**Figures 7**, **8**).

The average ASVs were highest in the Treatment 1 at 6 months (275 ± 20), indicating substantial fungal community richness under untreated healthy conditions. Conversely, the lowest average ASVs occurred in Treatment 5 (152 ± 52), highlighting a reduction in fungal diversity potentially due to the dominance or selective pressure exerted by *Trichoderma* spp. without biochar. Notably, *Trichoderma* spp. combined with biochar (Treatment 9) showed higher diversity (257 ± 29) compared to pure *Trichoderma* spp. alone, suggesting biochar’s role in promoting or maintaining fungal diversity. Diseased treatments with *Trichoderma* spp. showed similar values, with Treatment 8 at 209 ± 10 and Treatment 12 only slightly lower at 207 ± 10.

Analysis of species richness using the Chao1 index revealed consistent trends. The highest richness was recorded for the Treatment 1 at 6 months (270.523 ± 17.802), while Treatment 5 again displayed the lowest richness (152.733 ± 53.348). Treatment 9 recorded a relatively high richness (242.623 ± 22.767), reinforcing biochar’s role in sustaining a diverse microbial community. The diseased treatments showed intermediate richness, with Treatment 8 at 192.917 ± 10.852 and Treatment 12 slightly higher at 198.690 ± 9.133, indicating a partial recovery of fungal richness despite disease pressure, especially in the presence of biochar.

The Shannon diversity index, which accounts for both abundance and evenness of species, was highest in Treatment 2 (5.010 ± 0.075), illustrating a balanced fungal community. Among *Trichoderma* spp. treatments, Treatment 5 exhibited relatively higher diversity (4.227 ± 0.032) than Treatment 9 (3.393 ± 0.320). However, the lowest Shannon diversity was seen in Treatment 8, the diseased treatment that received pure *Trichoderma* spp. application (2.397 ± 0.249), suggesting significant fungal community imbalance under combined disease and pure *Trichoderma* spp. stress. In contrast, Treatment 12 (3.550 ± 0.312), the diseased treatment amended with T-mix showed slightly better fungal diversity, highlighting biochar’s positive impact even under disease stress.

Across all soil treatments, the fungal community was dominated by a few genera, while many others remained at low relative abundances. *Penicillium* and *Aspergillus*, both saprophytic fungi, were highly dominant in Treatment 1 at 0 months, comprising 40–58% and 5–17% of sequences, respectively. In Treatment 1 at 6 months, these genera continued to prevail with *Penicillium* spp. at 23% and *Aspergillus* spp. at 14% on average, indicating that *Penicillium* spp. and *Aspergillus* spp. thrived in the absence of amendments.

*Trichoderma* spp. itself became a major component in the communities where it was added. At 6 months, *Trichoderma* spp. reached 7–11% relative abundance in Treatment 5, compared to less than 1% in control soils. Treatment 9 also showed high *Trichoderma* spp. (averaging 6–8%), confirming successful establishment of the introduced biocontrol fungus. Another genus that proliferated in amended soils was *Talaromyces* spp. In Treatment 9, *Talaromyces* spp. became the most dominant genus, surging to 60% of sequences in that group. This extreme increase suggests that biochar may have created conditions favouring *Talaromyces* spp. Other putatively beneficial fungi also responded to the amendments. The arbuscular mycorrhizal genus *Paraglomus* spp. showed a notable rise in Treatment 11, reaching 0.04%, versus near-zero in other treatments. Although still a low percentage, this suggests that biochar might have created microsites favourable to AMF.

*Fusarium* spp. was detected at modest levels in these soils. In Treatment 1 at 0 months, *Fusarium* spp. averaged 0.6%. After 6 months, Treatment 1 saw *Fusarium* spp. increase to an average of 3.8%, suggesting that in the absence of interventions, *Fusarium* spp. may proliferate over time. Notably, treatments with Trichoderma had much lower *Fusarium* spp. levels. In Treatment 5, *Fusarium* spp. remained around 1.0%, and in Treatment 9 it was 1.6%, indicating a suppression relative to the control. This pattern implies that *Fusarium* spp. was either directly antagonised by *Trichoderma* spp. or indirectly kept in check by the altered community in those treatments.

*Ganoderma* spp. had surprisingly low representation in the amplicon data. *Ganoderma* spp. sequences were present only at trace levels, at less than 0.05% across all treatments, including in Treatments 3, 7 and 11, where it was intentionally introduced. Even in Treatment 3, *Ganoderma* spp. averaged less than 0.01%. However, in all *Ganoderma* spp.-inoculated soils, *Phanerodontia* spp., a wood-decay *Basidiomycete* genus, became overwhelmingly dominant. In Treatment 3, *Phanerodontia* spp. comprised 62% of the fungal community, and similarly 40–69% in Treatments 7 and 11. *Phanerodontia* spp. is a genus of white-rot fungus capable of decomposing lignin, much like *Ganoderma* spp. Its dominance suggests that it likely colonised the rubber wood blocks used to introduce *Ganoderma* spp., effectively outcompeting or preying upon *Ganoderma* spp. there. Consequently, *Ganoderma* spp. itself failed to establish a large population in soil, being displaced by this competitor. The introduction of *Trichoderma* spp. did not prevent the bloom of *Phanerodontia* spp. as even with *Trichoderma* spp. present, *Phanerodontia* spp. remained above 40%. This indicates a robust competitive advantage of *Phanerodontia* spp. on that woody substrate. Ecologically, this finding underscores that native or opportunistic saprophytes can suppress an introduced pathogen’s spread. *Ganoderma* spp. presence did have some community-level effects, as *Ganoderma* spp.-inoculated soils generally showed a reduction in overall fungal richness. However, this may instead have been likely due to *Phanerodontia* spp. monopolising resources. Additionally, certain opportunists like *Aspergillus* spp. and *Penicillium* spp. were relatively lower in *Ganoderma* spp.-challenged soils, presumably because the heavy colonisation of *Phanerodontia* spp. and other wood-rot fungi limited their growth. Minor pathogens did not show a consistent rise due to *Ganoderma* spp. If anything, the pathogen introduction paradoxically led to specific fungal genera, namely white-rot decomposers, dominating rather than typical soil pathogens.

The results demonstrate that introducing *Trichoderma* spp., with or without biochar, profoundly alters the soil fungal community composition. *Trichoderma* spp. are well-known as aggressive mycoparasites and competitors in soil, which feeds on other fungi by secreting cell wall-degrading enzymes and antibiotic compounds, thereby naturally limiting the proliferation of fungi like *Fusarium* spp. and *Rhizoctonia* spp. that might otherwise flourish (Muter et al, 2017). In this study, *Fusarium* spp. was indeed much lower in *Trichoderma* spp.-amended treatments than in the control, consistent with the antagonistic activity of *Trichoderma*spp. This aligns with the broader literature where *Trichoderma* spp. are widely used as biocontrol agents against soil-borne pathogens, not only reducing disease incidence but also often improving plant growth and health (Yao et al., 2023). *Trichoderma* spp. can colonise the rhizosphere and produce growth-promoting metabolites and hormones, enhance nutrient availability, and induce systemic resistance in plants (Yao et al., 2023). The significant increase of *Trichoderma* spp. relative abundance from less than 1% to more than 7% in treated soils confirms that the biocontrol inoculum established itself successfully. A recent study by Hang et al., 2022 similarly found that adding *Trichoderma* spp. in a bio-organic fertiliser shifted the resident fungal community in favour of indigenous growth-promoting fungi, which was correlated with enhanced plant biomass. In this case, the reduction of opportunistic fungi such as *Penicillium* spp. and *Aspergillus* app., and slight enrichment of beneficial taxa such as AMFs and *Mortierella* spp. in *Trichoderma* spp.-treated soils echoes that finding, suggesting that *Trichoderma* spp. not only directly antagonises pathogens, but can also steer the soil microbiome toward a more beneficial state (Wang et al, 2019).

Biochar is also observed to play an important modulatory role alongside *Trichoderma* spp. For instance, one experiment demonstrated that combining *T. harzianum* with biochar improved plant growth and greatly reduced pathogen wilt incidence compared to either amendment alone (Ahmad et al, 2024). In this experiment, Treatment 9, which was the treatment of *Trichoderma* spp. with biochar, maintained *Trichoderma* spp. abundance and overall fungal diversity at levels comparable to unamended soil, whereas Treatment 5, which was the treatment with a pure *Trichoderma* spp. application, tended to reduce diversity. This suggests biochar buffered the competitive exclusion effect of *Trichoderma* spp., perhaps by providing extra niche space for other fungi to persist. This is supported by a recent study that found that biochar in field soil was colonised predominantly by *Paraglomerales* spp., with this fungus accounting for about 78% of AMF sequences in biochar pores (Neuburger et al, 2024). This finding of increased *Paraglomus* spp. in Treatment 9 mirrors this phenomenon and implies that biochar may enhance mycorrhizal establishment. From an ecological perspective, the *Trichoderma* spp.–biochar combination appears to promote a more functionally balanced fungal community. This synergistic outcome aligns with growing evidence that coupling biochar with biocontrol fungi is a promising strategy for sustainable soil management (Ahmad et al, 2024).

The Tmix used in Treatment 11 provides a plausible steering intervention, as *Trichoderma* spp. alongside biochar can restructure microbial habitats and resources, often increasing microbial diversity and rebalancing communities, mechanisms that can indirectly limit pathogen expansion and support plant health (Elad et al., 2010; Xu et al., 2023). As such, Treatment 11 fostered a more functionally resilient fungal community that coexisted with *Ganoderma* spp. but maintained greater diversity and evenness, traits repeatedly linked to suppression of further invasion and stability under disease pressure (Wittebolle et al., 2009; van Elsas et al., 2012; Allison & Martiny, 2008; Xu et al., 2023; Chapelle et al., 2016; Jost, 2006; Chao et al., 2014).

## 5 Conclusion

In conclusion, a combination of chemical fertiliser, *Trichoderma* spp. and biochar, T-mix is the most effective combination for developing young oil palms roots. *Trichoderma* spp. is effective in controlling the effect of *Ganoderma* spp., be it in its liquid form or in the form of T-mix. However, T-mix would be easier to store and transport as it is in solid form and did not require any adjuvants such as stabilisers and chemicals. The shade house experiment also shows that *Trichoderma* spp. can be a useful plant growth stimulator, with the *Trichoderma* spp. treatments far outperforming all the other treatments as well as the absolute control. Further studies can be useful to understand the mechanisms responsible for the *Trichoderma* spp.-induced root development and rhizosphere community dynamics in diseased oil palms. To the authors knowledge, this is the first study to elucidate fungal community dynamics in *Trichoderma* spp.-biochar treatment of basal stem rot disease of oil palm.

## Data Availability

The datasets presented in this study can be found in online repositories. The names of the repository/repositories and accession number(s) can be found in the article/supplementary material.
